# Supplementary figures and images for: Functions of coastal feeder bluff systems: Implications for prioritizing protection and restoration
Source: PLoS One. 2025 Oct 15;20(10):e0334742. doi: 10.1371/journal.pone.0334742 (PMC12527195; doi:10.1371/journal.pone.0334742)

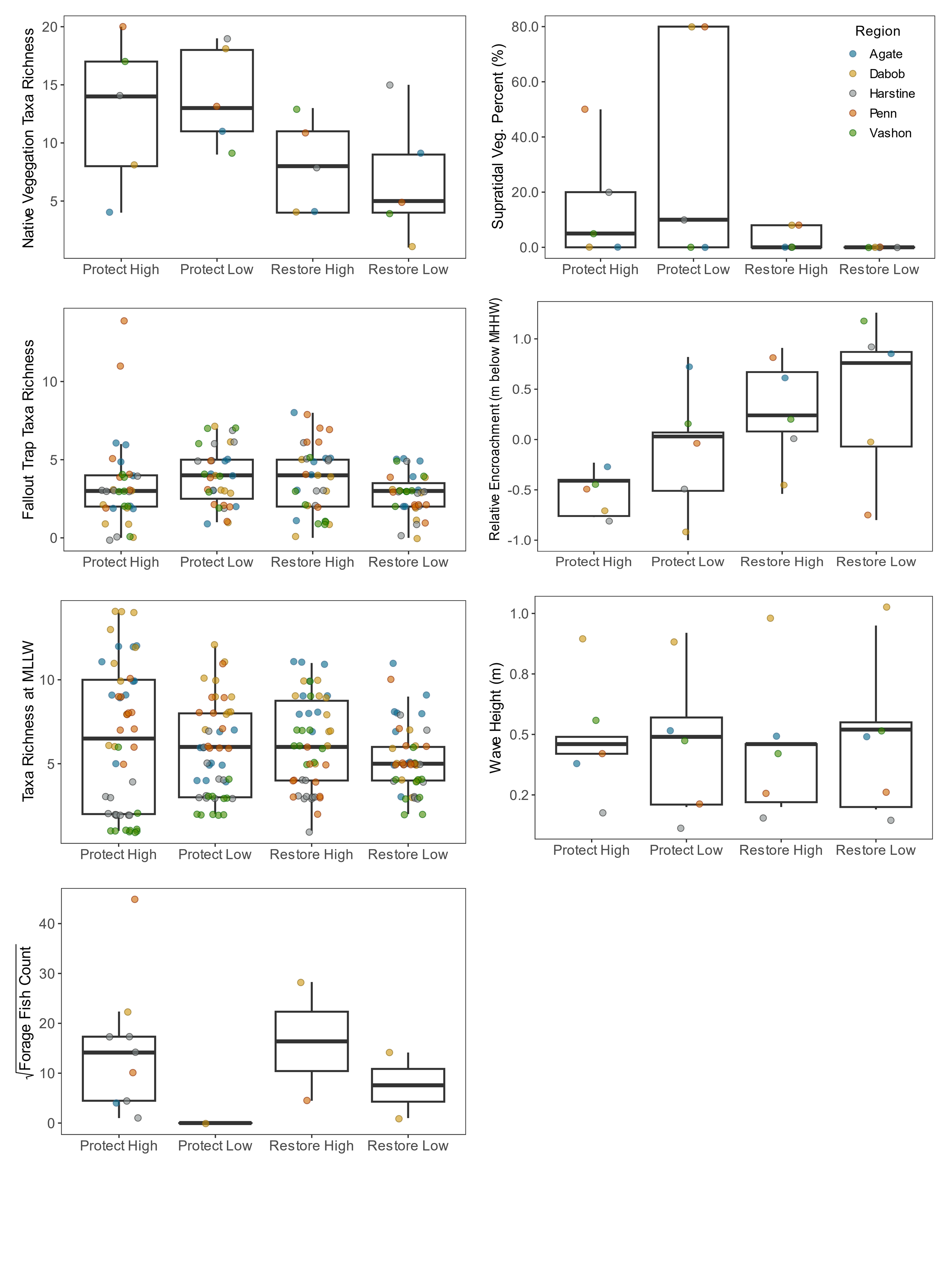

Supplement: S1 Fig — Data points are color-coded by Region, and are minimally staggered to avoid overlap. (TIF) [file pone.0334742.s001.tif]

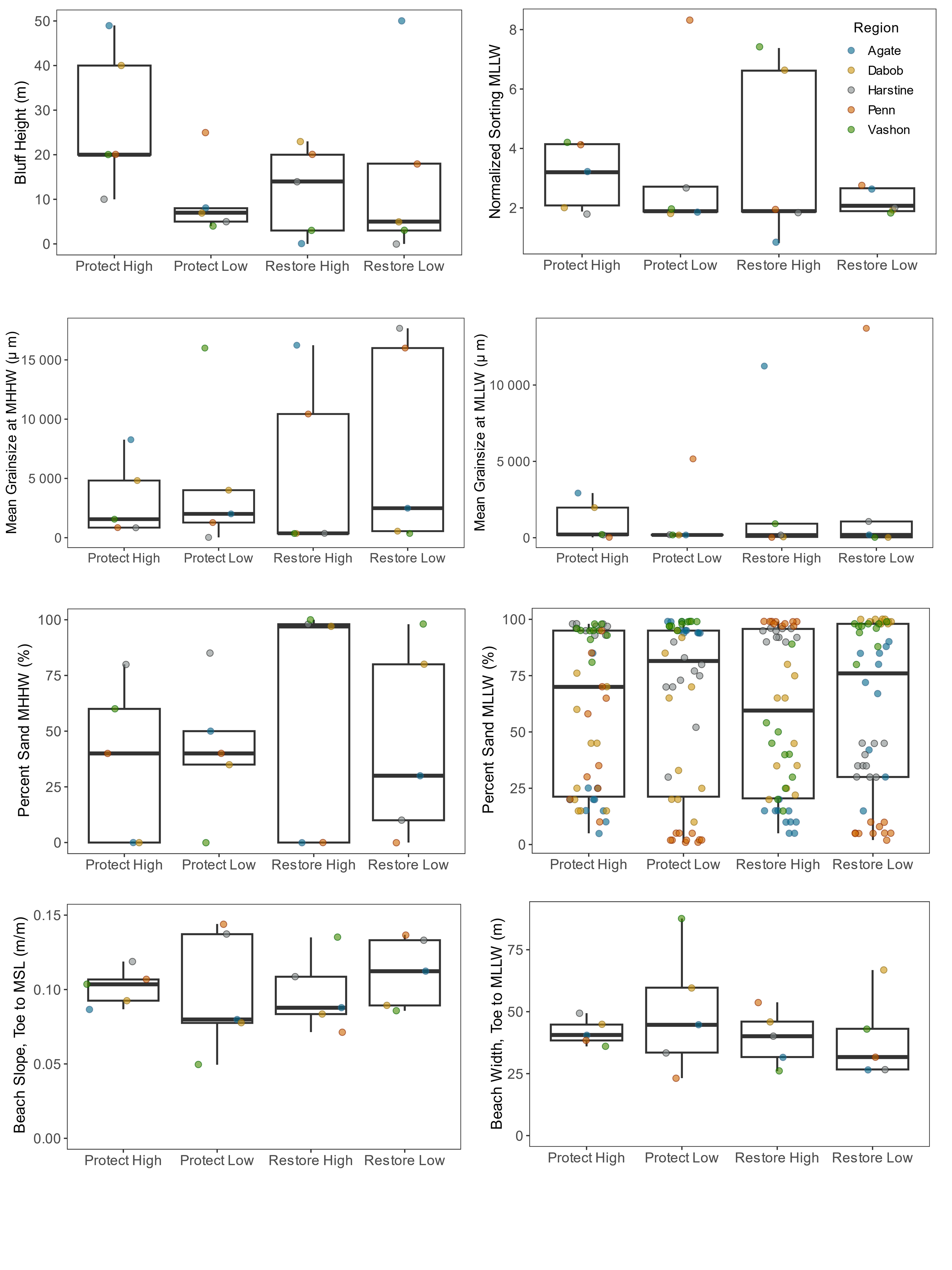

Supplement: S2 Fig — Data points are color-coded by Region, and are minimally staggered to avoid overlap. (TIF) [file pone.0334742.s002.tif]

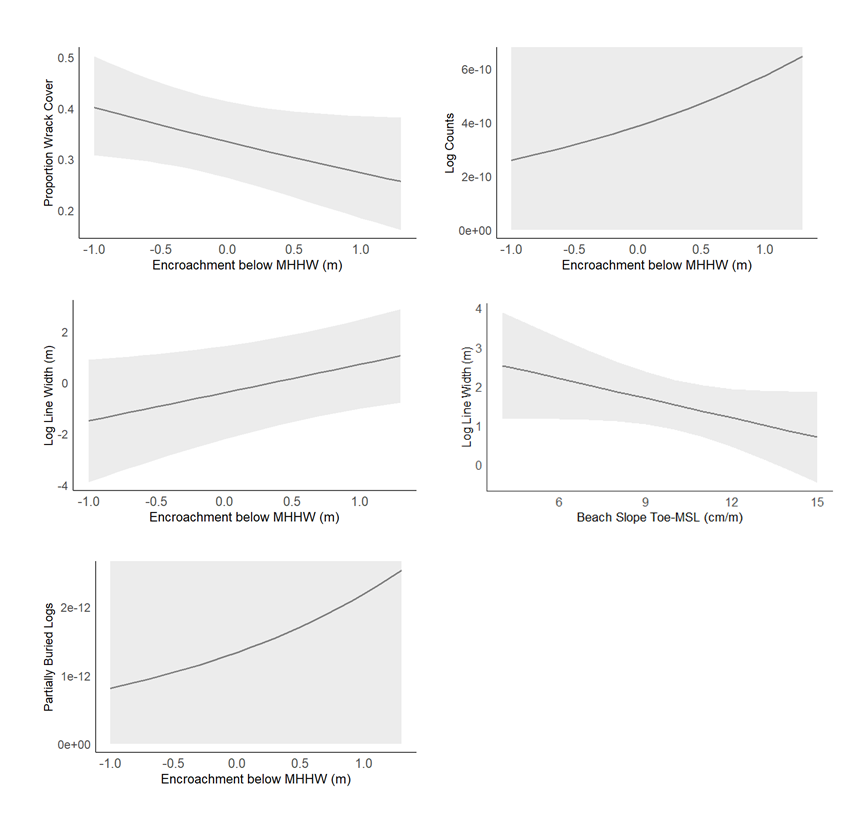

Supplement: S3 Fig — Response variables are proportion wrack cover, log line width, count of logs, count of partially buried logs, and log line width. 95% confidence intervals are shaded gray. (TIF) [file pone.0334742.s003.tif]

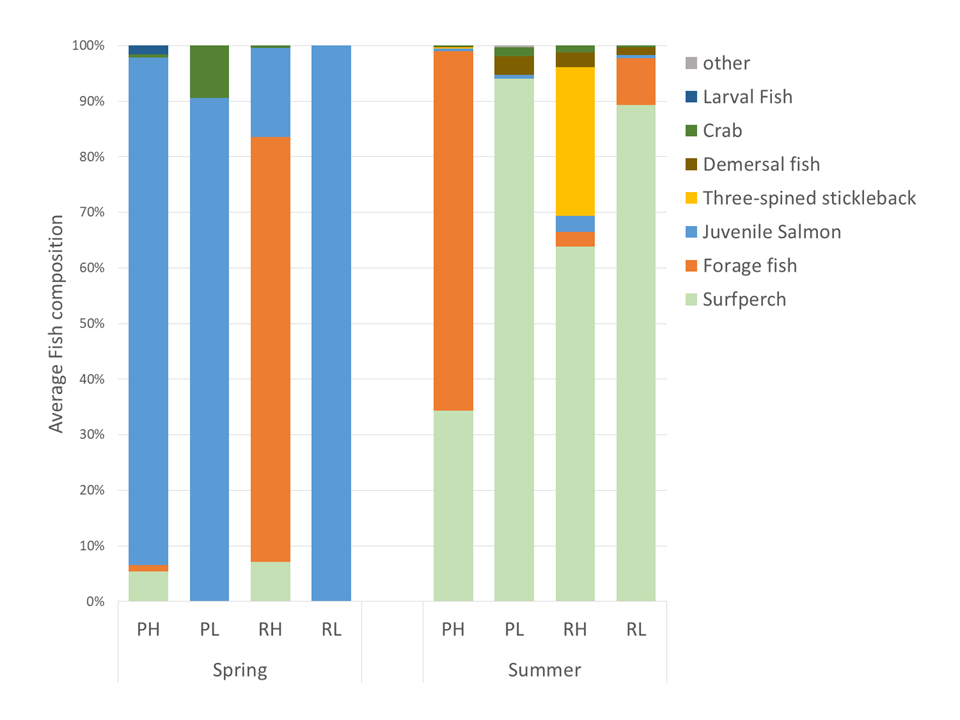

Supplement: S4 Fig — (TIF) [file pone.0334742.s004.tif]

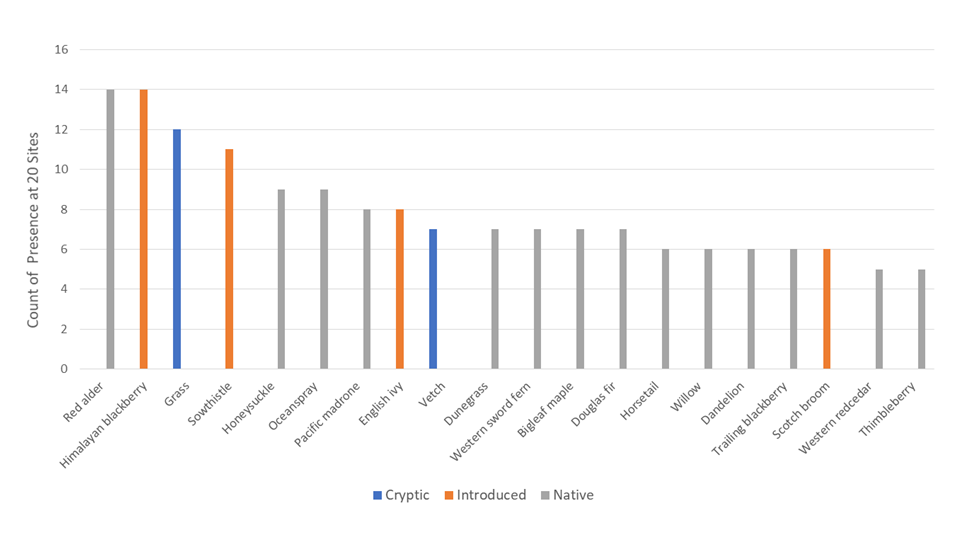

Supplement: S5 Fig — Colors show native, introduced, and cryptic status. (TIF) [file pone.0334742.s005.tif]

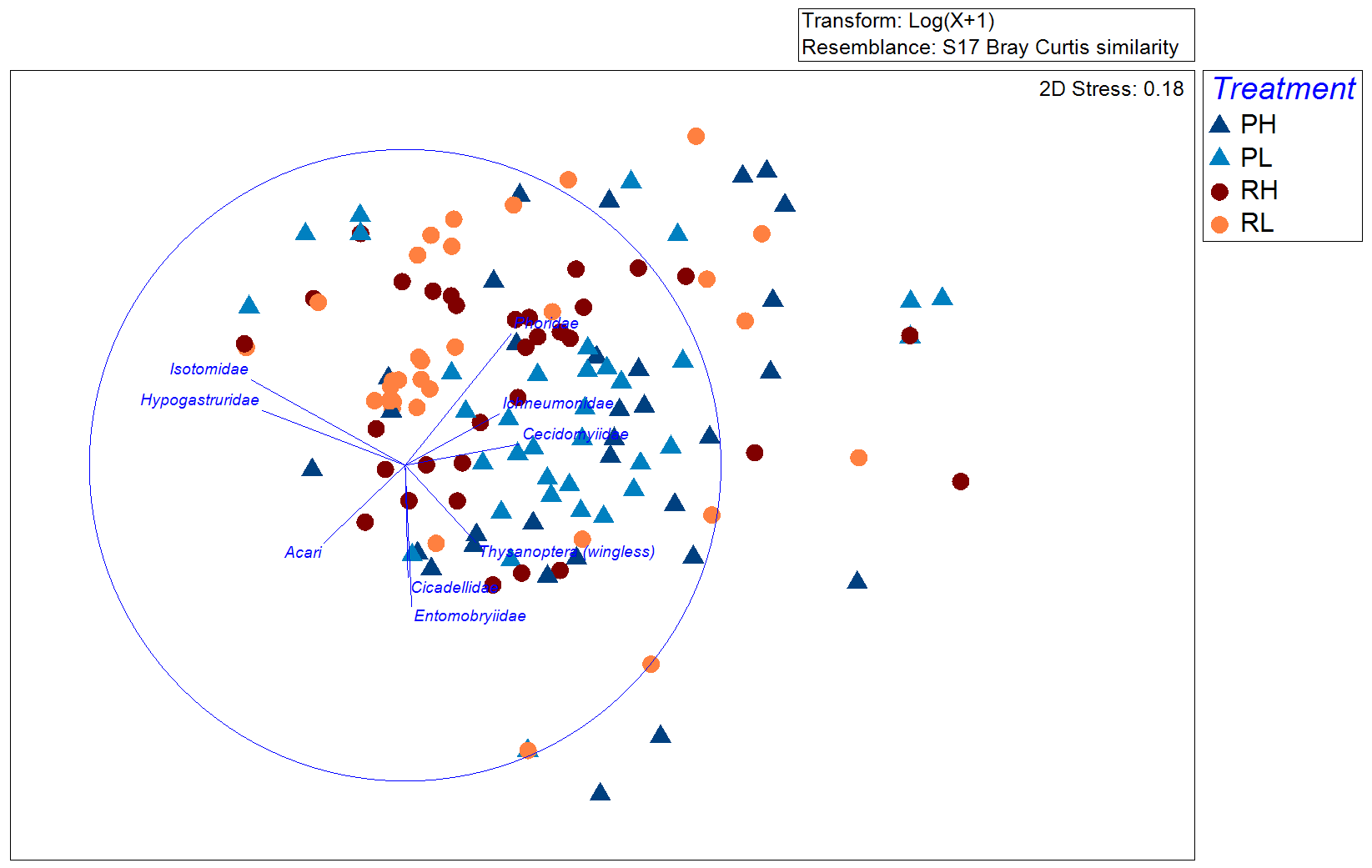

Supplement: S6 Fig — Symbols are the four treatments, with vectors of taxa correlation. PERMANOVA shows treatment is significant (p < 0.0001), with all pairwise comparisons different from each other (p < 0.05) except Protect High and Low. (TIF) [file pone.0334742.s006.tif]

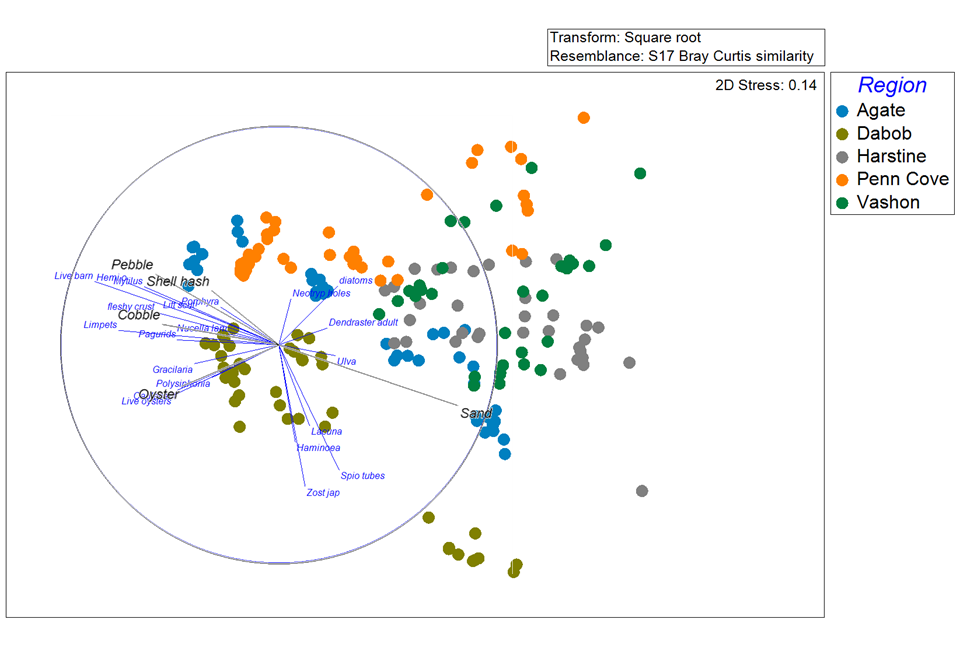

Supplement: S7 Fig — Vectors are taxa and sediment correlation. (TIF) [file pone.0334742.s007.tif]

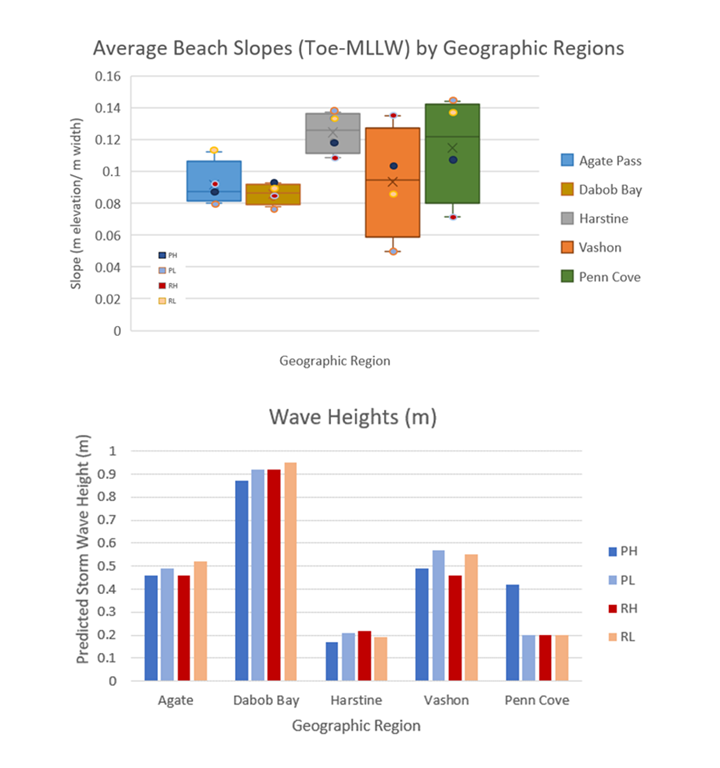

Supplement: S8 Fig — Visualized by geographic region and treatment. (TIF) [file pone.0334742.s008.tif]
