# Supplementary material for: Functions of coastal feeder bluff systems: Implications for prioritizing protection and restoration
Source: PLoS One. 2025 Oct 15;20(10):e0334742. doi: 10.1371/journal.pone.0334742 (PMC12527195; doi:10.1371/journal.pone.0334742)
Supplement: Attachment — Submitted filename: Response to Reviewers.docx [file pone.0334742.s016.docx]

PONE-D-25-22073

Functions of coastal feeder bluff systems: implications for prioritizing protection and restoration

PLOS ONE

Dear Dr. Toft,

Thank you for submitting your manuscript to PLOS ONE. After careful consideration, we feel that it has merit but does not fully meet PLOS ONE’s publication criteria as it currently stands. Therefore, we invite you to submit a revised version of the manuscript that addresses the points raised during the review process.

Two reviewers have provided helpful feedback for your revisions. The majority of the reviewers' recommendations focus on improving clarity and providing adequate context and other information for readers to follow you through to your conclusions. One reviewer suggested shortening the article. To clarify, PLOS does not have length restrictions but does encourage presenting information concisely.

Please submit your revised manuscript by Sep 07 2025 11:59PM. If you will need more time than this to complete your revisions, please reply to this message or contact the journal office at [plosone@plos.org](mailto:plosone@plos.org). When you're ready to submit your revision, log on to [https://www.editorialmanager.com/pone/](https://urldefense.com/v3/__https:/www.editorialmanager.com/pone/__;!!K-Hz7m0Vt54!noX437EjRWCjBVzrVIyUBer07NQ8UfKkaSAM84U5fZqYA7hsPx3seHsFSsBcxRXgxVJ4EVr9PqdIbw$) and select the 'Submissions Needing Revision' folder to locate your manuscript file.

Please include the following items when submitting your revised manuscript:

- A rebuttal letter that responds to each point raised by the academic editor and reviewer(s). You should upload this letter as a separate file labeled 'Response to Reviewers'.
- A marked-up copy of your manuscript that highlights changes made to the original version. You should upload this as a separate file labeled 'Revised Manuscript with Track Changes'.
- An unmarked version of your revised paper without tracked changes. You should upload this as a separate file labeled 'Manuscript'.

If you would like to make changes to your financial disclosure, please include your updated statement in your cover letter. Guidelines for resubmitting your figure files are available below the reviewer comments at the end of this letter.

If applicable, we recommend that you deposit your laboratory protocols in [protocols.io](http://protocols.io/) to enhance the reproducibility of your results. Protocols.io assigns your protocol its own identifier (DOI) so that it can be cited independently in the future. For instructions see: [https://journals.plos.org/plosone/s/submission-guidelines#loc-laboratory-protocols](https://urldefense.com/v3/__https:/track.editorialmanager.com/CL0/https:*2F*2Fjournals.plos.org*2Fplosone*2Fs*2Fsubmission-guidelines*23loc-laboratory-protocols/1/010f01983d6a5aa0-14ea9286-53cb-41f0-b613-e5b1d92fda43-000000/FcsU7OUDOqhx_KKUveG2EliuJzzBiSZJfT1RJjhgokI=221__;JSUlJSUl!!K-Hz7m0Vt54!noX437EjRWCjBVzrVIyUBer07NQ8UfKkaSAM84U5fZqYA7hsPx3seHsFSsBcxRXgxVJ4EVoP_TtAPA$). Additionally, PLOS ONE offers an option for publishing peer-reviewed Lab Protocol articles, which describe protocols hosted on [protocols.io](http://protocols.io/). Read more information on sharing protocols at [https://plos.org/protocols?utm_medium=editorial-email&utm_source=authorletters&utm_campaign=protocols](https://urldefense.com/v3/__https:/track.editorialmanager.com/CL0/https:*2F*2Fplos.org*2Fprotocols*3Futm_medium=editorial-email*26utm_source=authorletters*26utm_campaign=protocols/1/010f01983d6a5aa0-14ea9286-53cb-41f0-b613-e5b1d92fda43-000000/1B09Ss4egC5y4kWZitWC6eZeKPQSmtLV9WOsSViu_p4=221__;JSUlJSUl!!K-Hz7m0Vt54!noX437EjRWCjBVzrVIyUBer07NQ8UfKkaSAM84U5fZqYA7hsPx3seHsFSsBcxRXgxVJ4EVoM_uqcog$).

We look forward to receiving your revised manuscript.

Kind regards,

Stephanie S. Romanach, Ph.D.
Academic Editor
PLOS ONE

*Thank you for the helpful comments from yourself and both reviewers. We are pleased that the reviews were overall positive, and feel that the revised manuscript has been improved in clarity and context by reviewer input. Specific comments are addressed below:*

Journal Requirements:

When submitting your revision, we need you to address these additional requirements.

1. Please ensure that your manuscript meets PLOS ONE's style requirements, including those for file naming. The PLOS ONE style templates can be found at

[https://journals.plos.org/plosone/s/file?id=wjVg/PLOSOne_formatting_sample_main_body.pdf](https://urldefense.com/v3/__https:/journals.plos.org/plosone/s/file?id=wjVg*PLOSOne_formatting_sample_main_body.pdf__;Lw!!K-Hz7m0Vt54!noX437EjRWCjBVzrVIyUBer07NQ8UfKkaSAM84U5fZqYA7hsPx3seHsFSsBcxRXgxVJ4EVqeyQlpMQ$) and

[https://journals.plos.org/plosone/s/file?id=ba62/PLOSOne_formatting_sample_title_authors_affiliations.pdf](https://urldefense.com/v3/__https:/track.editorialmanager.com/CL0/https:*2F*2Fjournals.plos.org*2Fplosone*2Fs*2Ffile*3Fid=ba62*2FPLOSOne_formatting_sample_title_authors_affiliations.pdf/1/010f01983d6a5aa0-14ea9286-53cb-41f0-b613-e5b1d92fda43-000000/lOOSEBtOcg6rPLOicCPaEaZO3fKf2SVyPS2AliD4ED4=221__;JSUlJSUlJQ!!K-Hz7m0Vt54!noX437EjRWCjBVzrVIyUBer07NQ8UfKkaSAM84U5fZqYA7hsPx3seHsFSsBcxRXgxVJ4EVquzcvM-Q$).

*Manuscript is formatted accordingly*

2. We note that Figure 1 in your submission contain [map/satellite] images which may be copyrighted. All PLOS content is published under the Creative Commons Attribution License (CC BY 4.0), which means that the manuscript, images, and Supporting Information files will be freely available online, and any third party is permitted to access, download, copy, distribute, and use these materials in any way, even commercially, with proper attribution. For these reasons, we cannot publish previously copyrighted maps or satellite images created using proprietary data, such as Google software (Google Maps, Street View, and Earth). For more information, see our copyright guidelines: [http://journals.plos.org/plosone/s/licenses-and-copyright](https://urldefense.com/v3/__http:/journals.plos.org/plosone/s/licenses-and-copyright__;!!K-Hz7m0Vt54!noX437EjRWCjBVzrVIyUBer07NQ8UfKkaSAM84U5fZqYA7hsPx3seHsFSsBcxRXgxVJ4EVr3Vl34AQ$).

We require you to either (1) present written permission from the copyright holder to publish these figures specifically under the CC BY 4.0 license, or (2) remove the figures from your submission:

a. You may seek permission from the original copyright holder of Figure 1 to publish the content specifically under the CC BY 4.0 license.

We recommend that you contact the original copyright holder with the Content Permission Form ([http://journals.plos.org/plosone/s/file?id=7c09/content-permission-form.pdf](https://urldefense.com/v3/__http:/journals.plos.org/plosone/s/file?id=7c09*content-permission-form.pdf__;Lw!!K-Hz7m0Vt54!noX437EjRWCjBVzrVIyUBer07NQ8UfKkaSAM84U5fZqYA7hsPx3seHsFSsBcxRXgxVJ4EVrXggwEeQ$)) and the following text:

“I request permission for the open-access journal PLOS ONE to publish XXX under the Creative Commons Attribution License (CCAL) CC BY 4.0 ([http://creativecommons.org/licenses/by/4.0/](https://urldefense.com/v3/__http:/creativecommons.org/licenses/by/4.0/__;!!K-Hz7m0Vt54!noX437EjRWCjBVzrVIyUBer07NQ8UfKkaSAM84U5fZqYA7hsPx3seHsFSsBcxRXgxVJ4EVrGgm53Uw$)). Please be aware that this license allows unrestricted use and distribution, even commercially, by third parties. Please reply and provide explicit written permission to publish XXX under a CC BY license and complete the attached form.”

Please upload the completed Content Permission Form or other proof of granted permissions as an "Other" file with your submission.

In the figure caption of the copyrighted figure, please include the following text: “Reprinted from [ref] under a CC BY license, with permission from [name of publisher], original copyright [original copyright year].”

 b. If you are unable to obtain permission from the original copyright holder to publish these figures under the CC BY 4.0 license or if the copyright holder’s requirements are incompatible with the CC BY 4.0 license, please either i) remove the figure or ii) supply a replacement figure that complies with the CC BY 4.0 license. Please check copyright information on all replacement figures and update the figure caption with source information. If applicable, please specify in the figure caption text when a figure is similar but not identical to the original image and is therefore for illustrative purposes only.

The following resources for replacing copyrighted map figures may be helpful:

USGS National Map Viewer (public domain): [http://viewer.nationalmap.gov/viewer/](https://urldefense.com/v3/__http:/viewer.nationalmap.gov/viewer/__;!!K-Hz7m0Vt54!noX437EjRWCjBVzrVIyUBer07NQ8UfKkaSAM84U5fZqYA7hsPx3seHsFSsBcxRXgxVJ4EVo2Dmh49A$)

The Gateway to Astronaut Photography of Earth (public domain): [http://eol.jsc.nasa.gov/sseop/clickmap/](https://urldefense.com/v3/__http:/eol.jsc.nasa.gov/sseop/clickmap/__;!!K-Hz7m0Vt54!noX437EjRWCjBVzrVIyUBer07NQ8UfKkaSAM84U5fZqYA7hsPx3seHsFSsBcxRXgxVJ4EVrTv3xPkA$)

Maps at the CIA (public domain): [https://www.cia.gov/library/publications/the-world-factbook/index.html](https://urldefense.com/v3/__https:/www.cia.gov/library/publications/the-world-factbook/index.html__;!!K-Hz7m0Vt54!noX437EjRWCjBVzrVIyUBer07NQ8UfKkaSAM84U5fZqYA7hsPx3seHsFSsBcxRXgxVJ4EVqE9zEk7A$) and [https://www.cia.gov/library/publications/cia-maps-publications/index.html](https://urldefense.com/v3/__https:/www.cia.gov/library/publications/cia-maps-publications/index.html__;!!K-Hz7m0Vt54!noX437EjRWCjBVzrVIyUBer07NQ8UfKkaSAM84U5fZqYA7hsPx3seHsFSsBcxRXgxVJ4EVpQbLDJSA$)

NASA Earth Observatory (public domain): [http://earthobservatory.nasa.gov/](https://urldefense.com/v3/__http:/earthobservatory.nasa.gov/__;!!K-Hz7m0Vt54!noX437EjRWCjBVzrVIyUBer07NQ8UfKkaSAM84U5fZqYA7hsPx3seHsFSsBcxRXgxVJ4EVpPAXv6_w$)

Landsat: [http://landsat.visibleearth.nasa.gov/](https://urldefense.com/v3/__http:/landsat.visibleearth.nasa.gov/__;!!K-Hz7m0Vt54!noX437EjRWCjBVzrVIyUBer07NQ8UfKkaSAM84U5fZqYA7hsPx3seHsFSsBcxRXgxVJ4EVom3CqBZw$)

USGS EROS (Earth Resources Observatory and Science (EROS) Center) (public domain): [http://eros.usgs.gov/#](https://urldefense.com/v3/__http:/eros.usgs.gov/*__;Iw!!K-Hz7m0Vt54!noX437EjRWCjBVzrVIyUBer07NQ8UfKkaSAM84U5fZqYA7hsPx3seHsFSsBcxRXgxVJ4EVpuW8s0Ow$)

Natural Earth (public domain): [http://www.naturalearthdata.com/](https://urldefense.com/v3/__http:/www.naturalearthdata.com/__;!!K-Hz7m0Vt54!noX437EjRWCjBVzrVIyUBer07NQ8UfKkaSAM84U5fZqYA7hsPx3seHsFSsBcxRXgxVJ4EVqAp3Uk0Q$)

*Figure 1 copyright material has been documented, with attributing sources of basemaps in the figure legend. The map was created in Esri ArcGIS, with guidelines for permitted use of static maps in academic publications documented online here:*

[*https://doc.arcgis.com/en/arcgis-online/reference/static-maps.htm*](https://doc.arcgis.com/en/arcgis-online/reference/static-maps.htm)

*In the legend for Figure 1 we have followed their guidelines, and added: “Map created in ArcGIS Pro 3.5, basemap sources: Esri, TomTom, Garmin, FAO, NOAA, USGS, (c) OpenStreetMap contributors, and the GIS User Community. Map image is the intellectual property of Esri and is used herein under license. Copyright © 2025 Esri and its licensors. All rights reserved.”*

3. If the reviewer comments include a recommendation to cite specific previously published works, please review and evaluate these publications to determine whether they are relevant and should be cited. There is no requirement to cite these works unless the editor has indicated otherwise.

*Not applicable*

4. Please review your reference list to ensure that it is complete and correct. If you have cited papers that have been retracted, please include the rationale for doing so in the manuscript text, or remove these references and replace them with relevant current references. Any changes to the reference list should be mentioned in the rebuttal letter that accompanies your revised manuscript. If you need to cite a retracted article, indicate the article’s retracted status in the References list and also include a citation and full reference for the retraction notice.

*We have reviewed our reference list, with no changes*

Reviewers' comments:

Reviewer's Responses to Questions

**Comments to the Author**

1. Is the manuscript technically sound, and do the data support the conclusions?

The manuscript must describe a technically sound piece of scientific research with data that supports the conclusions. Experiments must have been conducted rigorously, with appropriate controls, replication, and sample sizes. The conclusions must be drawn appropriately based on the data presented.

Reviewer #1: Yes

Reviewer #2: Partly

2. Has the statistical analysis been performed appropriately and rigorously?

Reviewer #1: Yes

Reviewer #2: Yes

3. Have the authors made all data underlying the findings in their manuscript fully available?

The [PLOS Data policy](https://urldefense.com/v3/__http:/track.editorialmanager.com/CL0/http:*2F*2Fwww.plosone.org*2Fstatic*2Fpolicies.action*23sharing/1/010f01983d6a5aa0-14ea9286-53cb-41f0-b613-e5b1d92fda43-000000/bl1Ha_RpkqvrmiPdUC6BfK4V0civLbe3XY4xNoBEuxM=221__;JSUlJSU!!K-Hz7m0Vt54!noX437EjRWCjBVzrVIyUBer07NQ8UfKkaSAM84U5fZqYA7hsPx3seHsFSsBcxRXgxVJ4EVrZfmntSQ$) requires authors to make all data underlying the findings described in their manuscript fully available without restriction, with rare exception (please refer to the Data Availability Statement in the manuscript PDF file). The data should be provided as part of the manuscript or its supporting information, or deposited to a public repository. For example, in addition to summary statistics, the data points behind means, medians and variance measures should be available. If there are restrictions on publicly sharing data—e.g. participant privacy or use of data from a third party—those must be specified.

Reviewer #1: Yes

Reviewer #2: Yes

4. Is the manuscript presented in an intelligible fashion and written in standard English?

PLOS ONE does not copyedit accepted manuscripts, so the language in submitted articles must be clear, correct, and unambiguous. Any typographical or grammatical errors should be corrected at revision, so please note any specific errors here.

Reviewer #1: Yes

Reviewer #2: Yes

5. Review Comments to the Author

Please use the space provided to explain your answers to the questions above. You may also include additional comments for the author, including concerns about dual publication, research ethics, or publication ethics. (Please upload your review as an attachment if it exceeds 20,000 characters)

Reviewer #1: The paper presents a comprehensive analysis of beach functions provided by cliffs of Puget Sound, WA, USA. The vast amount of data covering a wide range of beach functions was analyzed, which is particularly worth commendation, as it provided grounds for a genuinely in-depth study geared toward possible management strategies regarding sediment availability as one of key factors of sustainable coastal zone management. In this context the Authors determined four types of possible strategies, two of which describe the action priority (high vs. low) and two other the action type (protect – i.e. prevent armoring, and restore, i.e. discard armoring). The integration of multiple datasets and their subsequent joint analysis with consistent metrics can therefore be recommended for similar studies at other shores; the paper definitely deserves publication as source reference for such studies. However, it is focused solely on the Puget Sound, so the presented methodology cannot be readily implemented at other sites due to specific features and constraints of that basin. Consequently, the results gained herein need to be generalized for other sites in the discussion and conclusions sections before the paper can serve as valuable source reference. Thus, I recommend a minor revision of the manuscript. The specific issues to be amended are listed below.

*We thank the reviewer for their helpful comments, and have revised the manuscript accordingly, focusing as suggested on generalizing the results in the Discussion section and the specific concerns below.*

1. The Authors confine their reflections to two basic management options regarding sediment budgets at diverse locations at Puget Sound: leaving coastal bluffs undisturbed to secure sediment supply (protection) and discarding man-made structures to unblock that supply (restoration). However, I found no reflection on why some coastal units were armored – only in L. 46-47 coastal squeeze in UK is mentioned as detrimentally impacting beach functions there. I believe omission of the human factor and the concentration on ecological function in Puget Sound produces somewhat insufficient description of the study area. We do not know what human uses forced armoring projects (large investments?, land development?, harbor construction?, recreation?, etc.). This information is necessary to gain a more comprehensive understanding of the underlying main coastal zone management concepts in the study area.

*We have clarified information on the reasons for armor installation, at the start of paragraph 4 where armor and appropriate literature are introduced: “In Puget Sound, 29% of shorelines are armored (i.e., seawall, bulkhead, rip-rap) in order to stabilize banks and protect human infrastructure [19], similar to elsewhere along the U.S. coastline [20] and worldwide [21, 22].”*

2. One of such concepts is linked to potential implications of reestablishment of sediment supply by considering maximum permissible (annual) cliff retreat vs. the functions of hinterland. Is this concept included in the preparation of local coastal management plans/strategies?

*Yes, we have clarified as suggested at the start of the 2^nd^ to last paragraph of the Discussion: “Given our findings, we can offer some concrete examples of how restoration actions may incorporate additional features along feeder bluffs besides armor removal (along with related actions such as sediment nourishment and managed retreat), often a goal along Puget Sound shorelines [61] and elsewhere [62]”*

3. Human interventions did take into account local beach functions by concentrating the structures near higher beach segments ( L. 466-468). Taking into account the geomorphology of Puget Sound (short fetches) I believe this basin is dominated by tide-induced currents and wave action is a secondary source of energy. However, there are numerous cliffs, where the waves are a predominant driver of coastal change. Coastal protection/management is much more challenging at such sites and the armor must be more robust, embracing lower beach portions as well. Moreover, the Puget Sound cliffs are generally composed of coarse(r) grains, offering some resistance against migration/depletion. By contrast, there are multiple cliffs made predominantly of sand, sometimes with insertions of lenses of organic soils, or containing layers of tills, silts or clay that together can form very complicated ‘sandwiches’. Sustainable management of such cliffs and restoration of their beach functions appears to be much more difficult, as mere removal of armoring may often lead to unacceptable damages to hinterland and public protests. The Authors are asked to consider these aspects in their reflections in the discussion and conclusions sections and thus achieve a more general perspective.

*We have clarified these aspects of waves and sediments as suggested by the reviewer, adding text starting in paragraph 2 of the Discussion, on sediments: “More broadly, bluffs in many coastal systems can be composed of stratigraphic sequences resulting in failure planes that impact the rate of bluff recession and angle of the exposed bluff face [1, 2]. Different geologic sequences could therefore alter the need for armoring of the toe of the bluff for protection of human infrastructure. Although we do not address these factors, the glacial sediment exposed at our studied bluffs contained only limited evidence of failure planes due to organic or clay-rich sediment and generally had similar face exposures.”; and also on waves: “At the tidally fluctuating shoreline where the interaction between marine waters and the bluff toe occurs, wind-forced waves provide the primary contribution to erosive bed stress in Puget Sound [3].”*

4. The Authors seem to be preoccupied with ‘passive’ approach to sustainability by considering two basic types of action – protect/restore. I wonder why they did not consider beach fills as the means of recharging local sediment sources. Again, I suspect it is related to the geomorphology of Puget Sound, its short fetches and the resulting energy fluxes from oblique directions, where aggressive, shore-normal wave action events are rare (cf. L. 201-203). At the open coasts cliff maintenance is often done using beach fills, sometimes supported by low-crested breakwaters and/or groins, aimed at minimum overall seascape interference. In such instances either fully nature-based solutions are implemented, or permanent intervention remains minimal (and acceptable). I would expect acknowledging of the fact that such solutions do exist and can maintain/restore beach functions. This remarks is related to L. 58-60, where the sedimentation cycle is mentioned – please note that at open coasts the sediments are often carried offshore and lost forever. This aspect is also important to highlight to what extent fully nature-based solutions at Puget Sound are feasible (e.g. by sediment recycling with beach fills – if possible at Puget Sound this solution should minimize the costs of nourishments).

*As noted above for a similar comment on bluff retreat, we have specifically noted sediment nourishment as a viable option, as suggested at the start of the 2^nd^ to last paragraph of the Discussion: “Given our findings, we can offer some concrete examples of how restoration actions may incorporate additional features along feeder bluffs besides armor removal (along with related actions such as sediment nourishment and managed retreat), often a goal along Puget Sound shorelines [61] and elsewhere [62].” Also as suggested, we have noted the comment on open coast sediments: “Ultimately, sediment sinks can be located in the nearshore, e.g., low elevation accretion shoreforms where sediments are deposited, or can be carried offshore into deeper basins.”*

5. Following the previous point I found no precise information about the sources of sediment from bluffs – implicitly it becomes available due to marine processes of erosion. Given the morphology of Puget Sound (long and narrow, interconnected basins) I wonder whether wind directions favor the development of surges at the extremities of long embayments forming the Sound (and contribute to erosion events there)? Second, are heavy rains encountered in the area that can trigger local slides developing by generation of pore pressure in cliff massifs? Third, is there a significant role of ice freezing inside the massifs that can contribute to reduction of cliff stability during spring seasons? These pieces of information would be very helpful for foreign readers.

*As suggested, we have clarified these points, building off of the previous comments on waves, sediments and erosion, and aspects of fetch in the Discussion, and adding to paragraph 4 of the Discussion: “Additional factors that can influence the rate of bluff failure and erosion are sediment composition and stratigraphic layering that control pore water pressure, and bluff incisions leading to weakness during heavy precipitation and frost or ice heave. Although these factors can influence the grain size and slope relationship on our studied mixed sediment beaches, effects were not clearly evident.”*

Reviewer #2: The research topic and findings contribute valuable data to the field. However, several revisions are necessary to improve the clarity and effectiveness of the article in communicating the study’s rationale, goals, and results. Please consider addressing the following concerns:

*We thank the reviewer for their helpful comments, and have revised the manuscript accordingly, focusing as suggested on improving clarity and the specific concerns below.*

1. Article Length: While the manuscript contains detailed information, its overall length exceeds typical standards and resembles a project report. It is recommended to shorten the article to improve readability and maintain focus.

*As suggested, we have shortened certain sections where possible, often when one of the reviewer’s asked for clarification and/or there was some redundancy – see paragraphs 2 and 5 in the Introduction, and paragraph 2 in the Discussion, as good examples of shortening while incorporating reviewer’s suggestions. We also note that both reviewers suggested to add material in certain sections.*

2. Introduction Coherence: Certain sections of the introduction lack coherence, which affects readability. For example, the relationship between coastal squeeze, coastal erosion, and flooding (Lines 45–47) is not clearly articulated. Please revise this section to ensure a more logical and cohesive presentation of ideas.

*As suggested, we have made the Introduction more coherent by (1) clearly articulating the relationship in the noted example, (2) editing paragraphs 2 and 5 for clarity as addressed above in article length, and (3) incorporating suggestions by Reviewer 1 on reasons for armor placement and off-shore drift of sediments.*

3. Study Area Map (Figure 1): The current map does not clearly depict the beach locations. Replacing it with a clearer map, such as one based on Google Earth or a more legible base map with clearly marked sampling sites, would help readers better understand the geographical context of the study.

*We have clarified the map as suggested, making the map more legible by (1) adding separate panels for each region detailing treatment locations, (2) adding more place names of key waterways and cities, and (3) adding to the figure legend to make links to photos of beaches in Fig 2 and specific site locations detailed in S1 Table. As noted in the next comment, likely the review copy of the map in the pdf was not as clear as the high quality original.*

4. Figure Quality: The resolution of all figures should be improved to ensure better visual clarity and presentation quality.

*This appears to be due to the review copy of the figures created in the pdf, the resolution of original figures is high quality.*

5. Line 121 – Use of "Sound-wide": The term "Sound-wide" may be unclear to readers unfamiliar with the region. If it refers to Puget Sound or another specific location, please clarify by explicitly stating the geographic name (e.g., "Puget Sound-wide") to improve accessibility for a broader audience.

*Clarified as suggested to "across Puget Sound"*

6. Materials and Methods – Line 118: Please include the site selection methodology and the rationale for selecting the 30 metrics in two clearly separated sections. It is important to introduce the full set of metrics and explain the reasoning behind their selection before discussing the data collection process. Additionally, refer readers to Table 1 for the complete list of metrics at this point, so they are familiar with the variables being analyzed before proceeding further into the methods.

*Clarified as suggested – we first detail the site selection methodology, and subsequently detail the metrics before data collection. For the complete set of metrics and rationale, we refer to S2 Table which more completely details this information, saving Table 1 for the Results section.*

7. Line 122 – Reference to S1 Table and External Report: Referring readers to S1 Table and an external report for key methodological information is not ideal in an article that aims to independently present its methods, background, and results. It is recommended to revise the caption for S1 Table to include an explanation of how the different ranks and value ranges should be interpreted (e.g., which ranges are considered low, moderate, or high).

*We clarified these sentences to more clearly separate the information summarized in S1 Table on our study sites, and material generated in the Beach Strategies external report. We also revised the caption for S1 Table to more clearly explain the reported information.*

8. Line 182 – First Use of "MLLW": I believe this is the first instance where "MLLW" appears in the text. Please spell out the full term.

*Defined at first use in the previous paragraph.*

9. Line 206. Did the analysis of ecological functions account for the age of shoreline armoring at the selected sites? Specifically, was the timing of when each site was armored considered? Do you think this factor could influence the assessed value of beach functions, and if so, how?

*Age of placement of armor was not addressed, as this historical data is not consistently available across our study sites – data related to armor amount that we included in analyses were the relative encroachment of armor into the tidal frame, and the percent of bluffs armored in a drift cell in site rankings of process degradation. Other studies have also noted incorporating age since armoring, we added a sentence in the Discussion related to this: “Other aspects of armor that may affect the observed impact include time since placement and type of material [23]. These should be considered in future regional mapping and management of sediment supply and related ecological function.”*

10. Line 253. In Table 1, please spell out the abbreviations PH, PL, RH, and RL to ensure clarity for readers.

*In the Table 1 legend we added: “Treatment abbreviations are Protect High (PH), Protect Low (PL), Restore High (RH), and Restore Low (RL).”*

11. Line 552. While the analysis, comparison, and ranking presented in this study emphasize ecological functions and sediment supply across the selected sites, and explore the benefits of restoration approaches, it remains unclear what the existing shoreline armoring is currently protecting, what the key functions of the studied shorelines are, and why they are important. Were these structures originally installed to safeguard properties, infrastructure, or other assets along developed coastlines? Clarifying the current role of the armored bluffs, along with the potential costs and benefits of the recommended actions, such as armor removal or the implementation of green shoreline approaches, would help readers better understand the rationale behind the study’s recommendations.

*Reviewer 1 had a similar comment, to which we clarified information on the reasons for armor installation at the start of paragraph 4 in the Introduction where armor and appropriate literature are introduced: “In Puget Sound, 29% of shorelines are armored (i.e., seawall, bulkhead, rip-rap) in order to stabilize banks and protect human infrastructure [19], similar to elsewhere along the U.S. coastline [20] and worldwide [21, 22].” We further clarify this in the Discussion starting in the section the reviewer highlights by adding “Strategies aimed at removing armor at the base of bluffs, in cases when the protection of upland human infrastructure is not substantiated or can be alleviated, can anticipate a local restoration trajectory that approaches the function of natural bluffs, thus potentially doubling the pre-restoration ecological function.” This in turn links to the subsequent paragraphs on living shorelines, and development of the conceptual model on bluff features, shoreline structure, and ecological functions.*

12. As the authors also noted, the physical, hydrological, and ecological characteristics of the 20 selected sites can vary significantly. How was this variability addressed to reduce uncertainty and potential error in the comparison results? It is recommended that the authors add a dedicated section to discuss the methodological limitations of the study, including potential sources of error and uncertainty in the analysis.

*We address this variability in our results as described in our data analyses, by conducting generalized linear mixed models (GLMMs) including predictor variables of ecological response such as relative encroachment of the bluff, beach slope and width, and accounted for variability of region of Puget Sound, i.e., “Site location in Puget Sound (i.e., region) was incorporated as a random effect to account for variation among regions”. All statistical tests document model estimates and error values. We note specific additions from other reviewer comments that also address limitations of the study – addressing age of armor and type of material (Reviewer 2) and geomorphic considerations across Puget Sound and elsewhere worldwide (Reviewer 1). We further expand on the reviewer’s suggestion by adding another limitation in the Discussion section, in the same paragraph as the previous comment on shoreline armor: “We encourage more thorough documentation of functions through time given the dynamic nature of these bluff-beach systems, especially as related to any measurements of future restoration effectiveness and the limitations of our one year field study.”*

I hope these comments assist the authors in enhancing the presentation of their research outcomes.

Regards,

6. PLOS authors have the option to publish the peer review history of their article ([what does this mean?](https://urldefense.com/v3/__https:/track.editorialmanager.com/CL0/https:*2F*2Fjournals.plos.org*2Fplosone*2Fs*2Feditorial-and-peer-review-process*23loc-peer-review-history/1/010f01983d6a5aa0-14ea9286-53cb-41f0-b613-e5b1d92fda43-000000/pI7WRrUs3AN8nspblUaBSs8AmB22iS7-bYaA_jvqy9M=221__;JSUlJSUl!!K-Hz7m0Vt54!noX437EjRWCjBVzrVIyUBer07NQ8UfKkaSAM84U5fZqYA7hsPx3seHsFSsBcxRXgxVJ4EVr3VMqYHA$)). If published, this will include your full peer review and any attached files.


If you choose “no”, your identity will remain anonymous but your review may still be made public.


**Do you want your identity to be public for this peer review?** For information about this choice, including consent withdrawal, please see our [Privacy Policy](https://urldefense.com/v3/__https:/track.editorialmanager.com/CL0/https:*2F*2Fwww.plos.org*2Fprivacy-policy/1/010f01983d6a5aa0-14ea9286-53cb-41f0-b613-e5b1d92fda43-000000/6nvzt7Tb6xA0XgclvcFV6r1JCj4Hfwf8urEATWJFuL4=221__;JSUl!!K-Hz7m0Vt54!noX437EjRWCjBVzrVIyUBer07NQ8UfKkaSAM84U5fZqYA7hsPx3seHsFSsBcxRXgxVJ4EVoO483dGg$).

Reviewer #1: **Yes:** Grzegorz Różyński, Polish Academy of Sciences, Institute of Hydro-Engineering, Gdańsk, Poland

Reviewer #2: No

While revising your submission, please upload your figure files to the Preflight Analysis and Conversion Engine (PACE) digital diagnostic tool, [https://pacev2.apexcovantage.com/](https://urldefense.com/v3/__https:/track.editorialmanager.com/CL0/https:*2F*2Fpacev2.apexcovantage.com*2F/1/010f01983d6a5aa0-14ea9286-53cb-41f0-b613-e5b1d92fda43-000000/2ZhBHvNOfd1xTv4e75AMRBlX8xK8rwHYfe2cabm0BYA=221__;JSUl!!K-Hz7m0Vt54!noX437EjRWCjBVzrVIyUBer07NQ8UfKkaSAM84U5fZqYA7hsPx3seHsFSsBcxRXgxVJ4EVqMV3Ouew$). PACE helps ensure that figures meet PLOS requirements. To use PACE, you must first register as a user. Registration is free. Then, login and navigate to the UPLOAD tab, where you will find detailed instructions on how to use the tool. If you encounter any issues or have any questions when using PACE, please email PLOS at [figures@plos.org](mailto:figures@plos.org). Please note that Supporting Information files do not need this step.

*We have uploaded our figures to PACE, and submitted those versions*
